# Hematologic Cancers Among Patients With Type 2 Diabetes Prescribed GLP-1 Receptor Agonists

**DOI:** 10.1001/jamanetworkopen.2025.0802

**Published:** 2025-03-06

**Authors:** Omer S. Ashruf, Jasmin Hundal, Ali Mushtaq, David C. Kaelber, Faiz Anwer, Abhay Singh

**Affiliations:** 1 College of Medicine, Northeast Ohio Medical University, Rootstown; 2 Department of Hematology and Medical Oncology, Taussig Cancer Center, Cleveland Clinic, Cleveland, Ohio; 3 Department of Internal Medicine, Cleveland Clinic, Cleveland, Ohio; 4 Center for Clinical Informatics Research and Education, The MetroHealth System, Cleveland, Ohio; 5 Department of Internal Medicine, Case Western Reserve University, Cleveland, Ohio; 6 Department of Pediatrics, Case Western Reserve University, Cleveland, Ohio; 7 Department of Population and Quantitative Health Sciences, Case Western Reserve University, Cleveland, Ohio; 8 Cleveland Clinic Lerner College of Medicine, Case Western Reserve University, Cleveland, Ohio

## Abstract

This cohort study compares the risks of developing different hematologic cancers in patients with type 2 diabetes treated with glucagon-like peptide–1 receptor agonists (GLP-1RAs) compared with metformin and insulin.

## Introduction

Type 2 diabetes (T2D) and obesity have been identified as independent risk factors for various cancers, including hematologic cancers.^1^ Glucagon-like peptide–1 receptor agonists (GLP-1RA) have emerged as an effective treatment, offering glycemic control, weight reduction,^2^ and immune modulation,^3^ and are associated with lower cancer risk, specifically solid tumors.^4^ However, the association of GLP-1RA with hematologic cancers remains unexplored. This study aims to compare the risks of hematologic cancers in patients with T2D treated with GLP-1RA compared with metformin and insulin.

## Methods

This retrospective cohort study was conducted using TriNetX, a repository of aggregated electronic health record data of medical encounters for approximately one-quarter of the US population. The platform includes patient information from various age groups, racial and ethnic backgrounds, income levels, and insurance types. The data reviewed are a secondary analysis of existing data, do not involve intervention or interaction with human participants, and are deidentified per the standard defined in Section §164.514(a) of the Health Insurance Portability and Accountability Act Privacy Rule. More information is provided in the eMethods in Supplement 1.

Patients with T2D prescribed GLP-1RA, insulin, or metformin between April 30, 2005, and October 31, 2023, were identified. We excluded individuals prescribed any antidiabetic medications before T2D diagnosis to identify drug-naive patients, and those with prior diagnoses of hematologic cancers. Patients prescribed GLP-1RA (lixisenatide, albiglutide, dulaglutide, semaglutide, liraglutide, tirzepatide, or exenatide) comprised the experimental cohort. Additionally, patients prescribed exclusively insulins or metformin were identified. The primary outcome was the first diagnosis of hematologic cancer. Two analyses were performed: one comparing patients taking only GLP-1RA with those taking only metformin, and another comparing patients taking only GLP-1RA with only insulin (eFigure in Supplement 1).

The study groups were independently propensity matched using a 1:1 nearest neighbor greedy matching algorithm for demographic variables, weight status, body mass index (calculated as weight in kilograms divided by height in meters squared), diabetic complications, hemoglobin A_1C_, genetic susceptibility, cancer screenings, radiation, cytotoxic agents, antidiabetic agents, intensive care unit admissions, and adverse social determinants. All relevant codes are provided in the eTable in Supplement 1. The primary outcome was first diagnosis of hematologic cancer within 15 years of the index event (antidiabetic agent prescription). Using the in-built Advanced Analytics platform, hazard ratios (HRs) with 95% CIs and cumulative incidences were estimated using Cox proportional hazard and Kaplan-Meier survival analyses with censoring applied (eMethods in Supplement 1). Significance tests were 2-sided and paired, and statistical significance was set at *P* < .05.

## Results

We identified 1 601 334 patients (mean [SD] age, 62.5 [14.4] years; 751 026 female [46.9%]) with T2D, of whom 51 617 received exclusively GLP-1RA, 611 115 received metformin, and 938 602 received insulin (Table 1). The mean (SD) duration of GLP-1RA prescription was 485.4 (139.0) days. After matching, there were 47 716 patients in the GLP-1RA–insulin analysis and 50 590 in the GLP-1RA–metformin analysis. Compared with metformin, GLP-1RA use was associated with a statistically significantly lower risk of myelodysplastic syndromes (MDSs) (HR, 0.61; 95% CI, 0.42-0.89) and myeloproliferative neoplasms (MPNs) (HR, 0.67; 95% CI, 0.52-0.87). There was no significant difference in risk of any other hematologic cancer. Compared with insulin, GLP-1RA use was associated with a significantly lower risk of myeloid leukemia (HR, 0.39; 95% CI, 0.25-0.60), lymphoid leukemia (HR, 0.45; 95% CI, 0.30-0.68), non-Hodgkin lymphoma (HR, 0.42; 95% CI, 0.30-0.58), MDS (HR, 0.19; 95% CI, 0.11-0.35), MPN (HR, 0.50; 95% CI, 0.41-0.61), monoclonal gammopathy (HR, 0.68; 95% CI, 0.52-0.88), multiple myeloma (HR, 0.49; 95% CI, 0.31-0.76), and amyloidosis (HR, 0.52; 95% CI, 0.27-0.98). Across all hematologic cancers, GLP-1RA use was associated with 54% lower risk compared with insulin (Table 2).

**Table 1.** Patient Characteristics and Covariates After Propensity Matching of GLP-1RA and Metformin and GLP-1RA and Insulins Groups^a^

Cohort and characteristics	Patients, No. (% of cohort)	Standardized difference^b^
Cohort 1, GLP-1RA (n = 50 590) and cohort 2, metformin (n = 50 590)		
Demographics		
Age, mean (SD), y		
1	55.9 (12.8)	0.028
2	55.6 (13.7)	
Sex		
Male		
1	15 583 (30.80)	0.003
2	15 515 (30.70)	
Female		
1	32 091 (63.40)	0.009
2	32 318 (63.90)	
Race and ethnicity		
American Indian or Alaska Native		
1	188 (0.40)	0.011
2	155 (0.30)	
Asian		
1	1287 (2.50)	0.021
2	1124 (2.20)	
Black or African American		
1	9954 (19.70)	0.012
2	10 206 (20.20)	
Hispanic or Latino		
1	3621 (7.20)	0.017
2	3403 (6.70)	
Native Hawaiian or other Pacific Islander		
1	274 (0.50)	0.01
2	238 (0.50)	
White		
1	30 942 (61.20)	0.013
2	31 273 (61.80)	
Unknown race		
1	6052 (12.00)	0.022
2	5688 (11.20)	
Diagnosis		
Overweight and obesity		
1	25 385 (50.20)	0.01
2	25 641 (50.70)	
Morbid (severe) obesity		
1	15 522 (30.70)	0.002
2	15 571 (30.80)	
Body mass index^c^		
25-29		
1	3337 (6.60)	0.023
2	2369 (5.50)	
30-39		
1	10 822 (21.40)	0.01
2	10 624 (21.00)	
40-49		
1	10 207 (20.20)	0.026
2	9532 (18.90)	
Family history of other malignant neoplasms of lymphoid, hematopoietic, and related tissues		
1	73 (0.10)	0.001
2	71 (0.10)	
Genetic susceptibility to malignant neoplasm		
1	159 (0.30)	0.01
2	133 (0.30)	
Encounter for screening for malignant neoplasms		
1	18 493 (36.60)	0.018
2	18 055 (35.70)	
Personal history of malignant neoplasm		
1	2923 (5.80)	0.023
2	2661 (5.30)	
Complications of type 2 diabetes^d^		
1	8067 (15.90)	0.011
2	7758 (15.30)	
Persons with potential health hazards related to socioeconomic and psychosocial circumstances^e^		
1	2015 (4.00)	0.02
2	1824 (3.60)	
Procedure		
Radiation		
1	453 (0.90)	0.013
2	395 (0.80)	
Critical care services		
1	943 (1.90)	0.023
2	795 (1.60)	
Medication		
Cytotoxic agents^f^		
1	106 (0.20)	0.004
2	98 (0.20)	
Dipeptidyl peptidase–4 inhibitors		
1	192 (0.40)	0.003
2	183 (0.40)	
Sulfonylureas		
1	320 (0.60)	0.005
2	299 (0.60)	
Thiazolidinediones		
1	99 (0.20)	0.004
2	107 (0.20)	
Sodium-glucose cotransporter 2 inhibitors		
1	387 (0.80)	0.016
2	320 (0.60)	
α-Glucosidase inhibitors		
1	14 (<0.01)	0.005
2	10 (<0.01)	
Hemoglobin A_1C _values		
<9%		
1	28 850 (57.00)	0.008
2	29 040 (57.40)	
>9%		
1	2712 (5.40)	0.001
2	2720 (5.40)	
Cohort 1, GLP-1RA (n = 47 716) and cohort 2, insulins (n = 47 716)		
Demographics		
Age at index, mean (SD), y		
1	56.2 (12.8)	0.044
2	55.6 (14.2)	
Sex		
Male		
1	15 046 (31.50)	0.032
2	14 334 (30.00)	
Female		
1	29 806 (62.50)	0.05
2	30 954 (64.90)	
Race and ethnicity		
American Indian or Alaska Native		
1	166 (0.30)	0.005
2	179 (0.40)	
Asian		
1	1269 (2.70)	0.027
2	1071 (2.20)	
Black or African American		
1	9449 (19.80)	0.022
2	9862 (20.70)	
Hispanic or Latino		
1	3334 (7.00)	0.013
2	3177 (6.70)	
Native Hawaiian or other Pacific Islander		
1	271 (0.60)	0.016
2	215 (0.50)	
White		
1	29 038 (60.90)	0.027
2	29 662 (62.20)	
Unknown race		
1	5881 (12.30)	0.056
2	5026 (10.50)	
Diagnosis		
Overweight and obesity		
1	23 286 (48.80)	0.006
2	23 425 (49.10)	
Morbid (severe) obesity		
1	13 963 (29.30)	0.001
2	13 976 (29.30)	
Body mass index^c^		
25-29		
1	3186 (6.70)	0.022
2	2609 (5.60)	
30-39		
1	10 077 (21.10)	0.051
2	9106 (19.10)	
40-49		
1	9263 (19.40)	0.032
2	8475 (17.80)	
Family history of other malignant neoplasms of lymphoid, hematopoietic, and related tissues		
1	73 (0.20)	0.003
2	67 (0.10)	
Genetic susceptibility to malignant neoplasm		
1	150 (0.30)	0.017
2	108 (0.20)	
Encounter for screening for malignant neoplasms		
1	17 138 (35.90)	0.054
2	15 905 (33.30)	
Personal history of malignant neoplasm		
1	2916 (6.10)	0.036
2	2521 (5.30)	
Complications of type 2 diabetes^d^		
1	8021 (16.80)	0.015
2	7491 (15.70)	
Persons with potential health hazards related to socioeconomic and psychosocial circumstances^e^		
1	1869 (3.90)	0.03
2	1597 (3.30)	
Procedure		
Radiation		
1	440 (0.90)	0.017
2	364 (0.80)	
Critical care services		
1	936 (2.00)	0.016
2	833 (1.70)	
Medication		
Cytotoxic agents^f^		
1	105 (0.20)	0.001
2	108 (0.20)	
Dipeptidyl peptidase–4 inhibitors		
1	178 (0.40)	0.008
2	201 (0.40)	
Sulfonylureas		
1	298 (0.60)	0.012
2	346 (0.70)	
Thiazolidinediones		
1	92 (0.20)	0.012
2	69 (0.10)	
Sodium-glucose cotransporter 2 inhibitors		
1	373 (0.80)	0.011
2	420 (0.90)	
α Glucosidase inhibitors		
1	12 (<0.01)	0.003
2	10 (<0.01)	
Hemoglobin A_1C_ values		
<9%		
1	26 613 (55.80)	0.016
2	26 245 (55.00)	
>9%		
1	2669 (5.60)	0.001
2	2657 (5.60)	

Abbreviation: GLP-1RA, glucagon-like peptide–1 receptor agonist.

SI conversion factor: To convert hemoglobin A_1C_ to proportion of total hemoglobin, multiply by 0.01.

^a^ The status and presence of covariates was reported any time prior to the index event (GLP-1RA, insulin, or metformin prescription).

^b^ All study covariates met a standardized difference less than 0.1, indicating successful propensity matching between study groups.

^c^ Body mass index is calculated as weight in kilograms divided by height in meters squared.

^d^ Complications of type 2 diabetes were defined as individual encounter codes for type 2 diabetes with hyperosmolarity, ketoacidosis, kidney complications, ophthalmic complications, neurological complications, circulatory complications, diabetic arthropathy, skin complications, or oral complications.

^e^ Persons with potential health hazards related to socioeconomic and psychosocial circumstances were defined as patients with encounters for problems related to education or health literacy, employment, occupational or environment, housing, upbringing, social environment, or psychosocial circumstances.

^f^ Cytotoxic agents include actinomycines, anthracyclines, bleomycin, ixabepilone, mitomycin, plicamycin, or related substances.

**Table 2.** Incidence of Hematologic Cancers Among Patients Receiving GLP-1RA Compared With Metformin and Insulins^a^

Outcome	Patients, No. (%)		HR (95% CI)^b^	*P *value^c^
	GLP-1RA	Comparator drug		
GLP-1RA vs metformin				
Myeloid leukemia	26 (0.1)	41 (0.1)	1.18 (0.78-1.98)	.52
Lymphoid leukemia	32 (0.1)	56 (0.1)	1.32 (0.84-2.09)	.23
Non-Hodgkin lymphoma	48 (0.1)	82 (0.2)	1.19 (0.82-1.74)	.36
Myelodysplastic syndromes	13 (0.1)	38 (0.1)	0.61 (0.42-0.89)	.01
Myeloproliferative neoplasms	140 (0.3)	260 (0.5)	0.67 (0.52-0.87)	.002
Monoclonal gammopathy	87 (0.2)	143 (0.3)	1.23 (0.93-1.63)	.14
Multiple myeloma	27 (0.1)	46 (0.1)	1.38 (0.84-2.27)	.21
Primary amyloidosis	14 (0.1)	29 (0.1)	0.93 (0.48-1.81)	.83
All hematologic cancers	130 (0.3)	240 (0.5)	1.14 (0.91-1.43)	.24
GLP-1RA vs insulins				
Myeloid leukemia	25 (0.1)	95 (0.2)	0.39 (0.25-0.60)	<.001
Lymphoid leukemia	32 (0.1)	107 (0.2)	0.45 (0.30-0.68)	<.001
Non-Hodgkin lymphoma	46 (0.1)	157 (0.3)	0.42 (0.30-0.58)	<.001
Myelodysplastic syndromes	13 (0.1)	101 (0.2)	0.19 (0.11-0.35)	<.001
Myeloproliferative neoplasms	130 (0.3)	360 (0.8)	0.50 (0.41-0.61)	<.001
Monoclonal gammopathy	84 (0.2)	196 (0.4)	0.68 (0.52-0.88)	.004
Multiple myeloma	27 (0.1)	85 (0.2)	0.49 (0.31-0.76)	<.001
Primary amyloidosis	13 (0.1)	42 (0.1)	0.52 (0.27-0.98)	.04
All hematologic cancers	127 (0.3)	420 (0.9)	0.46 (0.37-0.56)	<.001

Abbreviations: GLP-1RA, glucagon-like peptide–1 receptor agonists; HR, hazard ratio.

^a^ Study groups were matched for age at antidiabetic agent prescription, sex, race, body mass index, status of overweight or obesity, family history, screening encounters, genetic susceptibility, complications of diabetes, radiation exposure, intensive care unit admission, prior antidiabetic agents, cytotoxic agents, and hemoglobin A_1C_.

^b^ Kaplan-Meier analysis was conducted to compare cumulative incidence rates between matched cohorts within a 15-year time window from index event.

^c^ Log-rank tests were used to determine the statistical significance of time-to-event differences.

## Discussion

The findings of this cohort study suggest that GLP-1RAs are associated with reduced risk of developing several hematologic cancers, particularly MDS and MPN, in patients with T2D. This reduction in risk may be mediated by weight loss, the immunomodulatory properties of GLP-1RAs, or both. These associations appear to be independent of glycemic control, potentially through the reduction of proinflammatory cytokines implicated in hematopoiesis dysregulation and the development of MDS and MPN.^5^ Metformin is suggested to have potential cancer protective effects,^6^ which could account for the equivocal findings for non-MDS/MPN cancers in our analysis. Nonetheless, our analysis suggests that GLP-1RAs represent a promising novel strategy for reducing cancer risk. Study limitations include reliance on encounter codes, residual confounding by indication, limited adjustment for multiple testing and sensitivity analysis, lack of age stratification, and dose-response relationships. Further prospective studies are needed to explore the biological pathways of GLP-1RAs in cancer prevention.

## Notes

Supplement 1. **eMethods.** Database description and statistical analysis **eFigure.** Study flow diagram **eTable.** Respective codes for demographics, diagnoses, procedures, medications, and laboratory values used in the platforms

Supplement 2.
Data Sharing Statement
