# Supplementary material for: Hematologic Cancers Among Patients With Type 2 Diabetes Prescribed GLP-1 Receptor Agonists
Source: JAMA Netw Open. 2025 Mar 6;8(3):e250802. doi: 10.1001/jamanetworkopen.2025.0802 (PMC11886721; doi:10.1001/jamanetworkopen.2025.0802)
Supplement: Supplement 1. — eMethods. Database description and statistical analysis eFigure. Study flow diagram eTable. Respective codes for demographics, diagnoses, procedures, medications, and laboratory values used in the platforms [file jamanetwopen-e250802-s001.pdf]

Supplemental Online Content

Ashruf OS, Hundal J, Mushtaq A, Kaelber DC, Anwer F, Singh A. Hematologic cancers among patients with type 2 diabetes prescribed GLP-1 receptor agonists. *JAMA Netw Open*. 2025;8(3):e250802. doi:10.1001/jamanetworkopen.2025.0802

eMethods. Database description and statistical analysis
eFigure. Study flow diagram
eTable. Respective codes for demographics, diagnoses, procedures, medications, and laboratory values used in the platform

This supplemental material has been provided by the authors to give readers additional information about their work.

© 2025 Ashruf OS et al. *JAMA Network Open*. This is an open access article distributed under the terms of the CC-BY-NC-ND License, which does not permit alteration or commercial use, including those for text and data mining, AI training, and similar technologies.

eMethods. Database description and statistical analysis

The TriNetX platform provides access to aggregated and de-identified electronic health record (EHR) data of approximately 100 million patients from 66 healthcare organizations across the United States. The data used in this study was collected from the TriNetX US Collaborative Network. TriNetX, LLC is compliant with the Health Insurance Portability and Accountability Act (HIPAA), the US federal law which protects the privacy and security of healthcare data. This retrospective study is exempt from informed consent. The data reviewed is a secondary analysis of existing data, does not involve intervention or interaction with human subjects, and is de-identified per the de-identification standard defined in Section §164.514(a) of the HIPAA Privacy Rule. The process by which the data is de-identified is attested to through a formal determination by a qualified expert as defined in Section §164.514(b)(1) of the HIPAA Privacy Rule. This formal determination by a qualified expert refreshed on December 2020. All analyses were collected and analyzed on November 29, 2024.

Most healthcare organizations that TriNetX provides EHR data from are large academic medical institutions with both inpatient and outpatient facilities at multiple locations, accounting for more than a quarter of the United States population. This includes data from hospitals, primary care facilities, and specialty treatment providers. Patients were identified using International Classification of Diseases, Tenth Revision, Clinical Modification (ICD-10-CM) diagnosis codes, Logical Observation Identifier Names and Codes (LOINC) laboratory codes, Current Procedural Terminology (CPT) procedure codes, and medical prescription normalized (RxNorm) codes. TriNetX spans over various geographic locations (22% Northeast, 16% Midwest, 39% South, 13% West, 10% Unspecified). The database encompasses a diverse range of age groups, racial and ethnic backgrounds. Self-reported sex (female, male), race and ethnicity data in TriNetX comes from the underlying clinical EHR systems of the contributing healthcare systems. TriNetX maps race and ethnicity data from the contributing healthcare systems to the following categories: (1) Race: Asian, American Indian or Alaskan Native, Black or African American, Native Hawaiian or Other, White, Unknown race; and (2) Ethnicity: Hispanic or Latino, Not Hispanic or Latino, Unknown Ethnicity. Also reported are income levels, and insurance types. These insurance types include commercial insurances, governmental insurance, Center of Medicare and Medicaid Service programs, Veterans Affairs insurance, self-pay or uninsured cases, worker compensation insurance, and others. The database solely contains EHR data and does not link to or provide information on claims data. Any data that may resemble claims information originates from the EHR system itself, which also serves the purpose of generating claims.

The statistical analysis for this study was conducted using the Advanced Analytics platform within TriNetX, employing Cox proportional hazards models, Kaplan-Meier survival analysis, and hazard ratio estimation to evaluate the association between GLP-1 receptor agonist (GLP-1RA) use and the incidence of hematologic malignancies in patients with type 2 diabetes mellitus (T2DM). Cox proportional hazards regression models was utilized for all outcomes to estimate hazard ratios (HRs) and 95% confidence intervals (CIs), adjusting for demographic variables, clinical comorbidities, and confounding factors, including diabetic complications and cancer-related variables. To ensure the validity of the proportional hazards assumption, the generalized

© 2025 Ashruf OS et al. *JAMA Network Open*. This is an open access article distributed under the terms of the CC-BY-NC-ND License, which does not permit alteration or commercial use, including those for text and data mining, AI training, and similar technologies.

Schoenfeld residuals approach was applied. This method evaluates time-dependent covariate effects by assessing the correlation of residuals with time, confirming that the proportional hazards assumption was met. Kaplan-Meier survival curves were generated to compare cumulative incidence rates between matched cohorts, and log-rank tests were used to determine the statistical significance of survival differences. Censoring was applied to account for variability in follow-up duration and death. Further, patients are censored when the last clinical encounter or fact in the patient's record is in the time window for analysis and if the patient has the outcome after the index event but before the start of the time window. Propensity score matching (1:1 nearest neighbor) balanced baseline characteristics across comparison groups to mitigate confounding. The statistical significance was determined using two-sided tests at a p-value threshold of <0.05.

© 2025 Ashruf OS et al. *JAMA Network Open*. This is an open access article distributed under the terms of the CC-BY-NC-ND License, which does not permit alteration or commercial use, including those for text and data mining, AI training, and similar technologies.

eFigure. Study flow diagram.

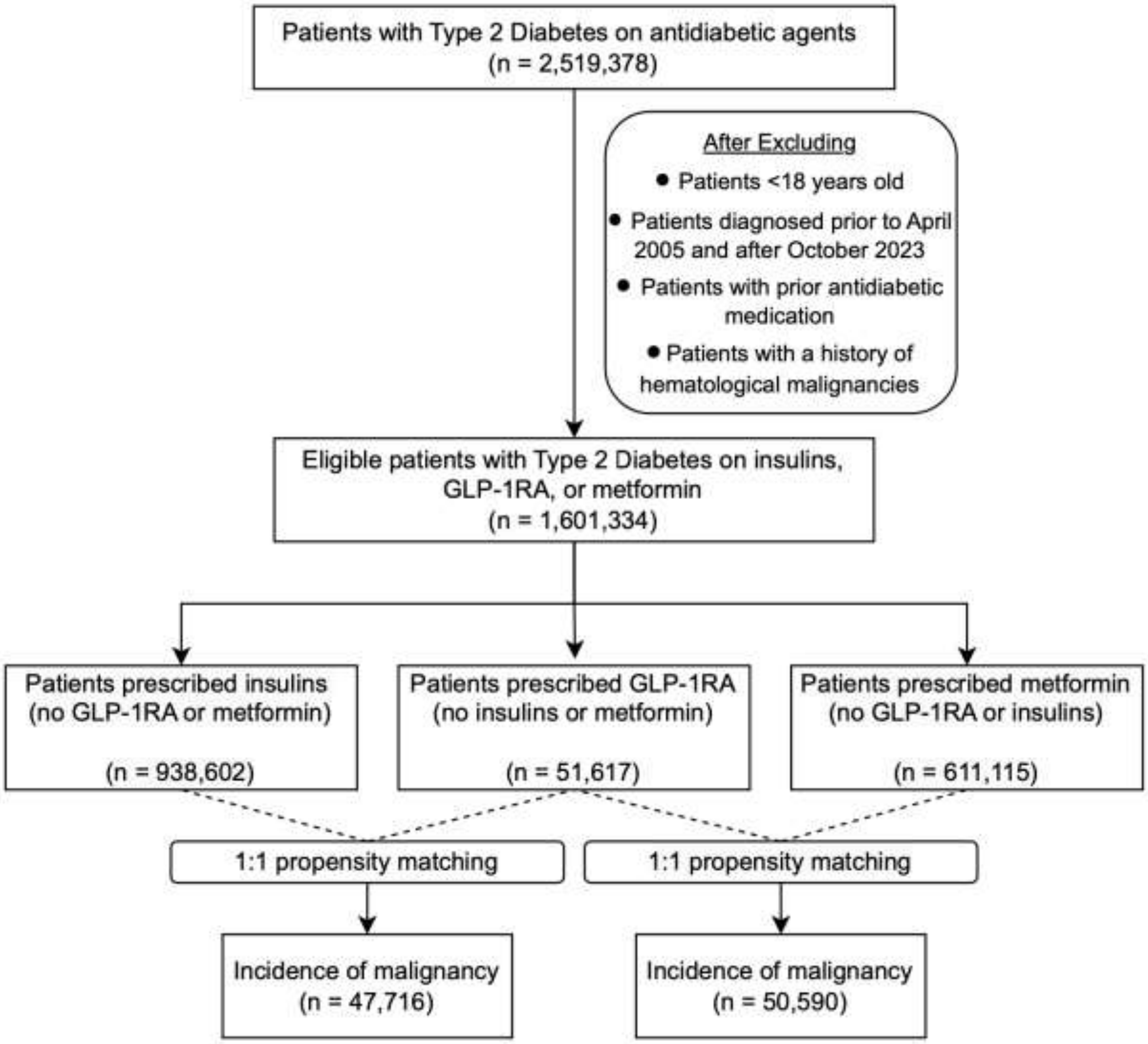

© 2025 Ashruf OS et al. *JAMA Network Open*. This is an open access article distributed under the terms of the CC-BY-NC-ND License, which does not permit alteration or commercial use, including those for text and data mining, AI training, and similar technologies.

eTable. Respective codes for demographics, diagnoses, procedures, medications, and laboratory values used in the platform.

	Code	Data Type
Demographics		
Age at index	Age	Continuous
Female	F	Present/Absent
Male	M	Present/Absent
Asian	Demographics: 2028-9	Present/Absent
American Indian or Alaska Native	Demographics: 1002-5	Present/Absent
Black or African American	Demographics: 2054-5	Present/Absent
Native Hawaiian or Other Pacific Islander	Demographics: 2076-8	Present/Absent
White	Demographics: 2106-3	Present/Absent
Other race	Demographics: 2131-1	Present/Absent
Hispanic/Latino	Demographics: 2186-5	Present/Absent
Unknown ethnicity	Demographics: UN	Present/Absent
Diagnoses		
Type 2 Diabetes	ICD-10: E11	Present/Absent
Type 2 diabetes mellitus with hyperosmolarity	ICD-10: E11.0	Present/Absent
Type 2 diabetes mellitus with ketoacidosis	ICD-10: E11.1	Present/Absent
Type 2 diabetes mellitus with kidney complications	ICD-10: E11.2	Present/Absent
Type 2 diabetes mellitus with ophthalmic complications	ICD-10: E11.3	Present/Absent
Type 2 diabetes mellitus with neurological complications	ICD-10: E11.4	Present/Absent
Type 2 diabetes mellitus with circulatory complications	ICD-10: E11.5	Present/Absent
Type 2 diabetes mellitus with diabetic arthropathy	ICD-10: E11.61	Present/Absent
Type 2 diabetes mellitus with skin complications	ICD-10: E11.62	Present/Absent
Type 2 diabetes mellitus with oral complications	ICD-10: E11.63	Present/Absent
Malignant neoplasms of lymphoid, hematopoietic and related tissue	ICD-10: C81-96<br><br>ICD-10: C81; Hodgkin lymphoma	Present/Absent

© 2025 Ashruf OS et al. *JAMA Network Open*. This is an open access article distributed under the terms of the CC-BY-NC-ND License, which does not permit alteration or commercial use, including those for text and data mining, AI training, and similar technologies.

	ICD-10:C82; Follicular lymphoma<br>ICD-10: C83; Non-follicular lymphoma<br>ICD-10: C84; Mature T/NK-cell lymphomas<br>ICD-10: C85; Other specified and unspecified types of non-Hodgkin lymphoma<br>ICD-10: C86; Other specified types of T/NK-cell lymphoma<br>ICD-10: C88; Malignant immunoproliferative diseases and certain other B-cell lymphomas<br>ICD-10: C90; Multiple myeloma and malignant plasma cell neoplasms<br>ICD-10: C91; Lymphoid leukemia<br>ICD-10: C92; Myeloid leukemia<br>ICD-10: C93; Monocytic leukemia<br>ICD-10: C94; Other leukemias of specified cell type<br>ICD-10: C95; Leukemia of unspecified cell type<br>ICD-10: C96; Other and unspecified malignant neoplasms of lymphoid, hematopoietic and related tissue<br>ICD-10: D47; Other neoplasms of uncertain behavior of lymphoid, hematopoietic and related tissue	
Overweight and Obesity	ICD-10: E66	Present/Absent
Morbid (severe) obesity due to excess calories	ICD-10: E66.01	Present/Absent
Body mass index [BMI] 25.0-25.9, adult	ICD-10: Z68.25	Present/Absent
Body mass index [BMI] 26.0-26.9, adult	ICD-10: Z68.26	Present/Absent
Body mass index [BMI] 27.0-27.9, adult	ICD-10: Z68.27	Present/Absent
Body mass index [BMI] 28.0-28.9, adult	ICD-10: Z68.28	Present/Absent
Body mass index [BMI] 29.0-29.9, adult	ICD-10: Z68.29	Present/Absent
Body mass index [BMI] 30-39, adult	ICD-10: Z68.3	Present/Absent
Body mass index [BMI] 40-44.9, adult	ICD-10: Z68.41	Present/Absent

© 2025 Ashruf OS et al. *JAMA Network Open*. This is an open access article distributed under the terms of the CC-BY-NC-ND License, which does not permit alteration or commercial use, including those for text and data mining, AI training, and similar technologies.

Body mass index [BMI] 45-49.9, adult	ICD-10: Z68.42	Present/Absent
Persons with potential health hazards related to socioeconomic and psychosocial circumstances	ICD-10: Z55-Z65<br><br>ICD-10: Z55; Problems related to education and literacy<br>ICD-10: Z56; Problems related to employment and unemployment<br>ICD-10: Z57; Occupational exposure to risk factors<br>ICD-10: Z58; Problems related to physical environment<br>ICD-10: Z59; Problems related to housing and economic circumstances<br>ICD-10: Z60; Problems related to social environment<br>ICD-10: Z62; Problems related to upbringing<br>ICD-10: Z63; Other problems related to primary support group, including family circumstances<br>ICD-10: Z64; Problems related to certain psychosocial circumstances<br>ICD-10: Z65; Problems related to other psychosocial circumstances	Present/Absent
Genetic susceptibility to malignant neoplasm	ICD-10: Z15.0	Present/Absent
Encounter for screening for malignant neoplasms	ICD-10: Z12	Present/Absent
Personal history of malignant neoplasm	ICD-10: Z85	Present/Absent
Family history of other malignant neoplasms of lymphoid, hematopoietic and related tissues	ICD-10: Z80.7	Present/Absent
Myeloid leukemia	ICD-10: C92; Myeloid leukemia<br>ICD-O-3: 9860/3; Myeloid leukemia, NOS	Present/Absent
Lymphoid leukemia	ICD-10: C91; Lymphoid leukemia<br>ICD-O-3: 9820/3; Lymphoid leukemia, NOS<br>ICD-O-3: 9823/3; B-cell chronic lymphocytic leukemia/small lymphocytic lymphoma	Present/Absent

© 2025 Ashruf OS et al. *JAMA Network Open*. This is an open access article distributed under the terms of the CC-BY-NC-ND License, which does not permit alteration or commercial use, including those for text and data mining, AI training, and similar technologies.

Myelodysplastic syndromes	ICD-10: D46; Myelodysplastic syndromes<br>ICD-10: C94.6; Myelodysplastic disease, not elsewhere classified<br>ICD-O-3: 998-999; Myelodysplastic syndromes	Present/Absent
Myeloproliferative neoplasms	ICD-10: D45; Polycythemia vera<br>ICD-10: D47.1; Chronic myeloproliferative disease<br>ICD-10: D47.3; Essential (hemorrhagic) thrombocythemia<br>ICD-10: D75.81; Myelofibrosis<br>ICD-O-3:995-996; Myeloproliferative neoplasms<br>ICD-O-3:9975/1; Myeloproliferative neoplasm, unclassifiable, uncertain whether benign or malignant	Present/Absent
Non-Hodgkin lymphoma	ICD-10: C82; Follicular lymphoma<br>ICD-10: C83; Non-follicular lymphoma<br>ICD-10: C85; Other specified and unspecified types of non-Hodgkin lymphoma<br>ICD-10: C84; Mature T/NK-cell lymphomas<br>ICD-O-3: 9591/3; Malignant lymphoma, non-Hodgkin, NOS<br>ICD-O-3: 9673/3; Mantle cell lymphoma<br>ICD-O-3: 9675/3; Malignant lymphoma, mixed small and large cell, diffuse<br>ICD-O-3: 9678/3; Primary effusion lymphoma<br>ICD-O-3: 9679/3; Mediastinal large B-cell lymphoma<br>ICD-O-3: 9680/3; Diffuse large B-cell lymphoma, NOS<br>ICD-O-3: 9684/3; Malignant lymphoma, large B-cell, diffuse, immunoblastic, NOS<br>ICD-O-3: 9687/3; Burkitt lymphoma, NOS<br>ICD-O-3: 9688/3; T-cell/histiocyte rich large B-cell lymphoma<br>ICD-O-3: 9689/3; Splenic marginal zone B-cell lymphoma	Present/Absent

© 2025 Ashruf OS et al. *JAMA Network Open*. This is an open access article distributed under the terms of the CC-BY-NC-ND License, which does not permit alteration or commercial use, including those for text and data mining, AI training, and similar technologies.

	ICD-O-3: 9690/3; Follicular lymphoma, NOS<br>ICD-O-3: 9691/3; Follicular lymphoma, grade 2<br>ICD-O-3: 9695/3; Follicular lymphoma, grade 1<br>ICD-O-3: 9698/3; Follicular lymphoma, grade 3<br>ICD-O-3: 9699/3; Marginal zone B-cell lymphoma, NOS<br>ICD-O-3: 9700/3; Mycosis fungoides<br>ICD-O-3: 9701/3; Sezary syndrome<br>ICD-O-3: 9702/3; Mature T-cell lymphoma, NOS<br>ICD-O-3: 9705/3; Angioimmunoblastic T-cell lymphoma<br>ICD-O-3: 9708/3; Subcutaneous panniculitis-like T-cell lymphoma<br>ICD-O-3: 9709/3; Cutaneous T-cell lymphoma, NOS<br>ICD-O-3: 9712/3; Intravascular large B-cell lymphoma<br>ICD-O-3: 9714/3; Anaplastic large cell lymphoma, T-cell and Null-cell type<br>ICD-O-3: 9715/3; Anaplastic large cell lymphoma, ALK negative<br>ICD-O-3: 9716/3; Hepatosplenic T-cell lymphoma<br>ICD-O-3: 9717/3; Intestinal T-cell lymphoma<br>ICD-O-3: 9718/3; Primary cutaneous anaplastic large cell lymphoma<br>ICD-O-3: 9719/3; NK/T-cell lymphoma, nasal and nasal type<br>ICD-O-3: 9724/3; Systemic EBV positive -cell lymphoproliferative disease of childhood<br>ICD-O-3: 9726/3; Primary cutaneous gamma-delta T-cell lymphoma<br>ICD-O-3: 9727/3; Precursor cell lymphoblastic lymphoma, NOS<br>ICD-O-3: 9728/3; Precursor B-cell lymphoblastic lymphoma<br>ICD-O-3: 9729/3; Precursor T-cell lymphoblastic lymphoma	

© 2025 Ashruf OS et al. *JAMA Network Open*. This is an open access article distributed under the terms of the CC-BY-NC-ND License, which does not permit alteration or commercial use, including those for text and data mining, AI training, and similar technologies.

Monoclonal gammopathy	ICD-10: D47.2; Monoclonal gammopathy<br>ICDO3:9765/1; Monoclonal gammopathy of undetermined significance, NOS	Present/Absent
Multiple myeloma	ICD-10: C90.0	Present/Absent
Primary amyloidosis	ICD-10: E85.4; Organ-limited amyloidosis<br>ICD-10: E85.81; Light chain (AL) amyloidosis	Present/Absent
Procedure		
Radiation	TNX Curated: 1001	Present/Absent
Critical Care Services	CPT: 1013729	Present/Absent
Medication		
Glucagon-like peptide-1 (GLP-1) analogues	ATC: A10BJ	Present/Absent
Sodium-glucose co-transporter 2 (SGLT2) inhibitors	ATC: A10BK	Present/Absent
Sulfonylureas	ATC: A10BB	Present/Absent
Thiazolidinediones	ATC: A10BG	Present/Absent
Alpha glucosidase inhibitors	ATC: A10BF	Present/Absent
Biguanides	ATC: A10BA	Present/Absent
Insulins and analogues	ATC: A10A<br>VA: HS501	Present/Absent
Cytotoxic antibiotics and related substances	ATC: L01D	Present/Absent
Laboratory		
Hemoglobin A1c	TNX Curated: 9037	Continuous

© 2025 Ashruf OS et al. *JAMA Network Open*. This is an open access article distributed under the terms of the CC-BY-NC-ND License, which does not permit alteration or commercial use, including those for text and data mining, AI training, and similar technologies.
