# Supplementary material for: Hematologic Cancers Among Patients With Type 2 Diabetes Prescribed GLP-1 Receptor Agonists
Source: JAMA Netw Open. 2025 Mar 6;8(3):e250802. doi: 10.1001/jamanetworkopen.2025.0802 (PMC11886721; doi:10.1001/jamanetworkopen.2025.0802)
Supplement: Supplement 2. — Data Sharing Statement [file jamanetwopen-e250802-s002.pdf]

Data Sharing Statement

Ashruf. Hematologic Cancers Among Patients With Type 2 Diabetes Prescribed GLP-1 Receptor Agonists. *JAMA Netw Open*. Published March 06, 2025.
doi:10.1001/jamanetworkopen.2025.0802

Data
Data available: No

Additional Information
Explanation for why data not available: The platform employed in this study aggregates de-identified patient data which is cloud-based and, therefore, can not be downloaded and/or exported. The platform also provides data in real-time, meaning the patient records shown are regularly updated and changed to reflect the most current diagnoses, procedures, medications, and so on. To mediate this, the authors provide specific time frames and network description to bring light to which dataset was used.
